# ACE (I/D) polymorphism and response to treatment in coronary artery disease: a comprehensive database and meta-analysis involving study quality evaluation

**DOI:** 10.1186/1471-2350-10-50

**Published:** 2009-06-04

**Authors:** Georgios Kitsios, Elias Zintzaras

**Affiliations:** 1 Department of Biomathematics, University of Thessaly School of Medicine, Larissa, Greece; 2 Center for Clinical Evidence Synthesis, Institute for Clinical Research and Health Policy Studies, Department of Medicine, Tufts Medical Center, Tufts University School of Medicine, 800 Washington Street, Tufts MC #63, Boston, MA 02111, USA

## Abstract

**Background:**

The role of angiotensin-converting enzyme (*ACE*) gene insertion/deletion (*I/D*) polymorphism in modifying the response to treatment modalities in coronary artery disease is controversial.

**Methods:**

PubMed was searched and a database of 58 studies with detailed information regarding *ACE I/D *polymorphism and response to treatment in coronary artery disease was created. Eligible studies were synthesized using meta-analysis methods, including cumulative meta-analysis. Heterogeneity and study quality issues were explored.

**Results:**

Forty studies involved invasive treatments (coronary angioplasty or coronary artery by-pass grafting) and 18 used conservative treatment options (including anti-hypertensive drugs, lipid lowering therapy and cardiac rehabilitation procedures). Clinical outcomes were investigated by 11 studies, while 47 studies focused on surrogate endpoints. The most studied outcome was the restenosis following coronary angioplasty (34 studies). Heterogeneity among studies (p < 0.01) was revealed and the risk of restenosis following balloon angioplasty was significant under an additive model: the random effects odds ratio was 1.42 (95% confidence interval:1.07–1.91). Cumulative meta-analysis showed a trend of association as information accumulates. The results were affected by population origin and study quality criteria. The meta-analyses for the risk of restenosis following stent angioplasty or after angioplasty and treatment with angiotensin-converting enzyme inhibitors produced non-significant results. The allele contrast random effects odds ratios with the 95% confidence intervals were 1.04(0.92–1.16) and 1.10(0.81–1.48), respectively. Regarding the effect of *ACE I/D *polymorphism on the response to treatment for the rest outcomes (coronary events, endothelial dysfunction, left ventricular remodeling, progression/regression of atherosclerosis), individual studies showed significance; however, results were discrepant and inconsistent.

**Conclusion:**

In view of available evidence, genetic testing of *ACE I/D *polymorphism prior to clinical decision making is not currently justified. The relation between *ACE *genetic variation and response to treatment in CAD remains an unresolved issue. The results of long-term and properly designed prospective studies hold the promise for pharmacogenetically tailored therapy in CAD.

## Background

Coronary artery disease (CAD), including its most severe complication, myocardial infarction (MI), is a complex disorder resulting from the interaction between genetic and environmental factors. Despite extensive efforts using the candidate gene approach or genome-wide linkage studies, the responsible molecular and genetic determinants remain largely unidentified [1,2]. Recently, genome-wide association studies provided more convincing evidence for CAD-associated genomic loci, generating cautious optimism for disentangling the disease pathophysiology and defining novel targets for treatment [3].

CAD mortality has been falling consistently in western countries, as a result of population-wide improvements in cardiovascular risk factors and modern cardiology treatments for CAD patients [4]. Nevertheless, a considerable inter-individual variability in response to the various treatment modalities for CAD, both invasive and pharmacological, has been described [5]. Given the large number of interventions currently available for the treatment and prevention of CAD and the large number of patients eligible to receive them, even small sources of variation in efficacy and safety have important implications for public health. An important source of variability in response to treatment is attributed to the patient's genetic profile [1,4,5].

Among the most studied genes for its implication in pathogenesis of CAD and related outcomes is angiotensin converting enzyme (*ACE*) gene, located on chromosome 17q23 [6-8]. The genetic polymorphism in intron 16, characterized by an *insertion *(*I*) or a *deletion *(*D*) of a 287 noncoding base pair Alu repeat sequence (dbSNP rs4646994) has been correlated with the levels of circulating, intracellular and heart tissue activity of ACE [9]. Apart from conferring susceptibility, the *ACE *gene has been also proposed to play a role in modifying the effect of various treatments in CAD. This potential modifying role has been investigated by numerous studies on several treatment-outcome settings. However, the available evidence published to date is weak, owing to sparseness of data or disagreements among studies. The aim of this study is to summarize the available *ACE I/D *and response-to-treatment studies for CAD and, where applicable, to quantify the effect size of the estimated risk associated with this polymorphism by meta-analysis.

## Methods

### Selection of studies

A comprehensive search of the PubMed database from its inception through March 2008 was conducted. We combined search terms for *ACE *genotype and CAD. Search terms included (ACE OR angiotensin converting enzyme) AND (gene OR polymorphism OR genetic variant) AND (myocardial infarction OR coronary artery disease OR coronary heart disease OR ischemic heart disease OR myocardial ischemia OR angina OR acute coronary syndrome). The retrieved studies were manually screened to assess their appropriateness for inclusion criteria. All references cited in the studies were also reviewed to identify additional published articles not indexed in the PubMed database. Case reports, editorials and review articles were excluded. The search was restricted to English-language articles of studies in humans.

The review included genetic association studies fulfilling the following inclusion criteria: (1) providing cases diagnosed with CAD or cohorts followed for CAD development, (2) using guideline-incorporated primary or secondary prevention measures for CAD [10-13], (3) investigating clinical outcomes of CAD (in primary or secondary prevention studies) or surrogate outcomes predicting clinical events in CAD patients, (4) providing information on genotype frequency for *ACE I/D *polymorphism or estimated genetic effects on response to treatment, and (5) using validated molecular methods for genotyping. Studies investigating susceptibility, progression, severity or survival, irrespective of treatment effect, were excluded from this review.

### Data extraction

Two investigators (GK and EZ) independently extracted data. The extracted data included information about the study design characteristics, the assessed outcomes, the cohort characteristics, the intervention used and finally, the reported results. Disagreements were resolved through consensus. The quality of each study was also critically assessed by reporting a composite quality score to allow comparison among studies.

### Data synthesis – Statistical analysis

For each study, the statistical significance of the main findings for each treatment-outcome group was recorded or the respective odds ratios were calculated by the extracted genotypic frequencies.

In the case of studies with similar outcome definition criteria, identical intervention and available genotype frequency for each group, a meta-analysis was performed (see results). Three meta-analyses were performed to investigate the association between *ACE I/D *and the risk of restenosis after angioplasty for the allele contrast (*D *vs *I*), the recessive (*DD *vs. *ID/II*), the dominant (*DD/ID *vs. *II*), the additive (*DD *vs *II*) and the co-dominant (*ID *vs *DD/II*) models. We calculated the overall odds ratio (OR) with the corresponding 95% confidence interval (CI) using the fixed effects (FE) and random effects (RE; DerSimonian and Laird) models. Statistical heterogeneity across the various studies was tested with the use of *Q*-statistic [14]. A p value < 0.10 indicated a significant statistical heterogeneity across studies, allowing for the use of RE model.

A cumulative and recursive cumulative meta-analysis was also carried out [6,14]. Cumulative and recursive cumulative meta-analyses provide a framework for updating a genetic effect from all studies and a measure of how much the genetic effect changes as evidence accumulates. Thus, cumulative meta-analysis indicates the trend in estimated risk effect and recursive cumulative meta-analysis indicates the stability in risk effect. In cumulative meta-analysis, studies were chronologically ordered by publication year, then, the pooled ORs were obtained at the end of each year, i.e. at each information step. In recursive cumulative meta-analysis, the relative change in pooled OR in each information step (pooled OR in next year/pooled OR in current year) was calculated. A differential magnitude of effect comparing large versus small studies for the allele contrast was verified using the Egger regression test [15].

In addition to the main (or overall) analysis which included all available data, a subgroup analysis for each "race" was also performed. 'Racial' descent was categorized into Caucasian descents (European and American whites), East Asian descents, mixed and populations of Turkish ancestry [14].

The impact of study quality was assessed by performing subgroup analysis on studies with high quality and low quality components. The following parameters were considered as quality components: A. Internal validity criteria: homogeneous study group, blindness of genotyping, registration of loss to follow up, genotyping procedure (original procedure or use of Insertion-specific primers) [16], genotyping replication with another protocol, blindness and objectiveness of angiograms assessment. B. Data description and analysis criteria: data by gender provided, power calculations provided, overlapping with previous studies, description of cases recruitment procedure, assessment of gene-gene interactions, control for possible clinical and other modifiers between genotypes, control of co-interventions that bear on outcome for each genotype, availability of data, appropriate statistics-description and discussion of possible genetic effects. High quality studies were defined as studies that exceeded the median quality score [6].

Analyses were performed using StatsDirect (StatsDirect Ltd), Compaq Visual Fortran90, and GLIM3.77.44–50 [6].

## Results

### Eligible studies

**Figure 1:**
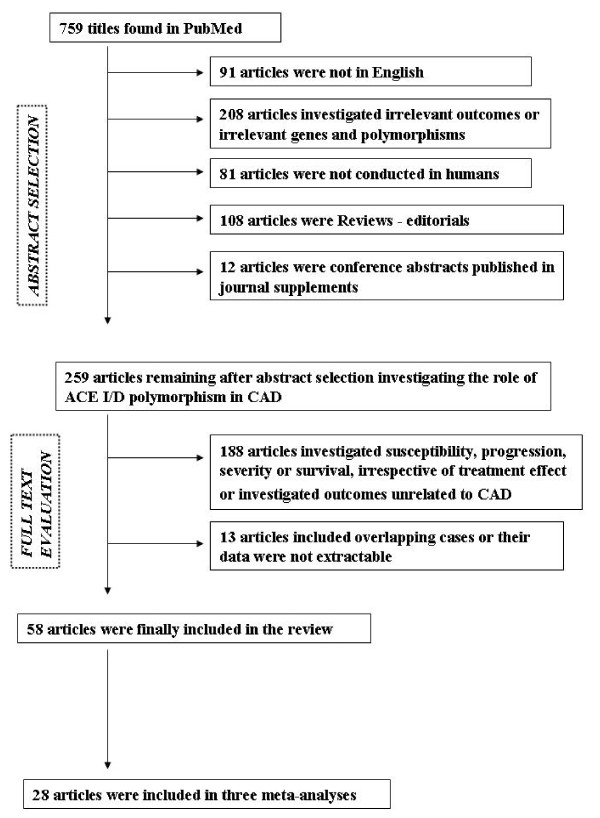
**Flow chart of retrieved studies and studies excluded, with specification of reasons**.

The literature search identified 759 citations. All citations identified through the literature search were independently screened by two investigators (GK and EZ) according to the inclusion criteria. Two hundred fifty-nine articles were retrieved and evaluated against the same criteria. Data from 58 articles [17-74] that investigated the association between *ACE I/D *polymorphism and response to treatment in CAD met the inclusion criteria, and were included in the review. Next, data from 28 studies [17-26,29-37,42-46,49] met the meta-analysis eligibility criteria and were included in the context of three meta-analyses. Figure 1 presents a flow chart of retrieved studies and studies excluded, with specification of reasons.

### Summary statistics

Details from studies included in our database are provided in Table 1 and Additional file 1. The interventions investigated were classified in two major categories: invasive (40 studies) and conservative (18 studies). The invasive treatments included coronary revascularization by percutaneous transluminal coronary angioplasty (PTCA) and coronary artery by-pass grafting (CABG). The conservative treatments included pharmacological interventions (16 studies) or cardiac rehabilitation procedures (two studies). The investigated outcomes were classified as clinical (11 studies) or surrogate (47 studies). The populations enrolled were of Caucasian (39 studies), East Asian (10 studies), mixed (six studies) or Turkish ancestry (three studies). Regarding study designs, our database included 44 cohort studies, eight randomized controlled trials, five cross-sectional and one retrospective study.

**Table 1:** Summary information of studies included in the meta-analyses.

**First author, Year, Country [ref]**	**Study design, duration of FU**	**Cohort description [No of patients (M/F), Ethnicity, Mean Age (SD), inclusion criteria]**	**Restenosis definition criteria**	**Intervention**	**Gene-gene interaction assessed? (gene)**	**Quality score**
				**Balloon angioplasty**		
Volzke, 2000, Germany [17]	cohort 6 months	511 (388/123), Caucasians, 60.6 (8.6), CAD patients undergoing elective PTCA of a previously untreated native coronary artery	> 50% progression of the residual stenosis at FU	PTCA-balloon	No	35
Yoshida, 1999, Japan [18]	cohort 5.21 (3.9) years	123 (nr), East Asians, 58.2 (10.2), MI patients undergoing PTCA discharged from hospital at the start of FU	nr	PTCA-balloon	No	30
Kasi, 1996, Spain [19]	cohort 6 months	69 (57/12), Caucasians, 58 (9.9), UA patients undergoing PTCA	diameter stenosis > 50% at FU	PTCA-balloon	No	27
Kamitani, 1995, Japan [20]	cohort 6 months	103 (103/00), East Asians, 52 (1), Primary PTCA for MI patients	> 50% progression of the residual stenosis at FU	PTCA-balloon	No	27
Samani, 1995, UK [21]	cohort 4 months	233 (194/39), nr, 56 (1), single-vessel PTCA in the Subcutaneous Heparin and Angioplasty Restenosis Prevention (SHARP) study	> 50% progression of the residual stenosis at FU	PTCA-balloon	No	34
van Bockxmeer, 1995, Australia [22]	cohort 6 months	207 (170/37), Caucasians, 57 (9), CAD patients undergoing elective PTCA	> 50% progression of the residual stenosis at FU	PTCA-balloon	Yes (*APOE*)	33
Tsukada, 1997, Japan [23]	cohort 3 months	96 (nr), East Asians, 60 (1.0), CAD patients undergoing elective PTCA	diameter stenosis > 50% at FU	PTCA-balloon	No	28
Beohar, 1995, USA [24]	cohort 3 months	89 (nr), Caucasians, 63.9 (10), CAD patients undergoing elective PTCA	diameter stenosis > 50% at FU	PTCA-balloon	No	24
Zee, 2001, Spain [25]	cohort 6 months	342 (305/37), Caucasians, 58.9 (9.6), CAD patients undergoing PTCA	> 50% progression of the residual stenosis at FU	PTCA-balloon	No	37
Ohishi, 1993, Japan [26]	cohort 6 months	82 (nr), East Asians, nr, MI patients undergoing primary PTCA	> 50% progression of the residual stenosis at FU	PTCA-balloon	No	23
Hamon, 1998, France [37]	cohort 6 months	271 (229/42), Caucasians, 60 (10), CAD patients undergoing PTCA	diameter stenosis > 50% at FU	PTCA-balloon	Yes (*AGT1R*)	34
				**Angioplasty with stent deployment**		
Amant, 1997, France [30]	cohort 6 months	146 (117/29), Caucasians, 60 (10) CAD patients undergoing PTCA	diameter stenosis > 50% at FU	PTCA-STENT	No	37
Wijpkema, 2006, Netherlands [29]	cohort 9 months	2888 (2050/838), Caucasians, 62 (11), CAD patients undergoing elective PTCA	death from cardiac causes, MI attributable to target vessel and target vessel revascularization	PTCA-STENT	Yes (*AGT, AGT1R, AGT2R, HMOX1*)	35
Gomma, 2002, UK [31]	cohort 6 months	205 (155/50), Caucasians, 59.4 (9.9), CAD patients undergoing PTCA	diameter stenosis > 50% at FU	PTCA-STENT	No	27
Ruy, 2002, Korea [32]	cohort 6 months	238 (178/60), East Asians, 59.5 (9.9), CAD patients undergoing PTCA	diameter stenosis > 50% at FU	PTCA-STENT	Yes (*CYP11B2, AGT*)	32
Ribichini, 2004, Italy [33]	cohort 6 months	897 (160/737), Caucasians, 61 (10), CAD patients undergoing PTCA	diameter stenosis > 50% at FU	PTCA-STENT	No	33
Taniguchi, 2001, Japan [34]	cohort 6 months	67 (50/17), East Asians, 65.2 (9.7), CAD patients undergoing PTCA	> 50% progression of the residual stenosis at FU	PTCA-STENT	No	23
Koch, 2000, Germany [35]	cohort 1 year	1850 (1458/392), Caucasians, 62.9 (10), CAD patients undergoing PTCA	diameter stenosis > 50% at FU	PTCA-STENT	No	40
Gurlek, 2000, Turkey [36]	cohort 6 months	132 (112/20), Turks, 53 (9), CAD patients undergoing PTCA	diameter stenosis > 50% at FU	PTCA-STENT	No	29
Guneri, 2005, Turkey [43]	cohort 9 months (2.9)	94 (59/35), Turks, 59.6 (9.9), CAD diabetic patients undergoing PTCA for stable angina pectoris	diameter stenosis > 50% at FU	PTCA-STENT	No	26
				**Angioplasty with ACEi treatment**		
Ribichini, 2003, Italy [42]	Cohort 6.3 (2.5) months	271 (nr), Caucasians, 61 (10), CAD patients undergoing PTCA	diameter stenosis > 50% at FU	PTCA-STENT + ACEi	No	35
Okamura, 1999, Japan [44]	cohort 6 months	97 (84/13), East Asians, 60 (2), CAD patients undergoing PTCA for stable angina pectoris	diameter stenosis > 50% at FU	PTCA-balloon + Imidapril 5 mg	No	29
Okumura, 2002, Japan [45]	cohort 6 months	92 (73/19), East Asians, 64.3 (8.9), CAD patients undergoing PTCA	diameter stenosis > 50% at FU	PTCA-STENT + Quinapril 18 mg	No	22
Ferrari, 2002, multicenter (Europe) [46]	cohort 6 months	154 (119/35), Caucasians, 61 (9.9), CAD patients undergoing PTCA	> 50% progression of the residual stenosis at FU	PTCA-STENT + ACEi	No	36
Jorgensen, 2001, Netherlands [49]	cohort 6 months	369 (293/76), Caucasians, 59 (43–73), CAD patients undergoing PTCA for stable angina	diameter stenosis > 50% at FU	PTCA-STENT + ACEi	No	40

Abbreviations: DCA: directional coronary atherectomy, FU: follow-up, UA: unstable angina, MI: myocardial infarction, RCT: randomized controlled trial, ACEi: angiotensin converting enzyme inhibitors, nr: non-reported

We now present the results for each intervention in turn.

### Invasive treatment strategies

#### Percutaneous transluminal coronary angioplasty

Since the first reports of successful angioplasty of coronary atherosclerotic lesions, restenosis has been encountered as a significant limitation to the long-term efficacy of the procedure. Restenosis is an angiographically-defined outcome correlated with reduction in the quality of life and increase in the morbidity or even the mortality of patients [75]. Stenting has rapidly become the preferred method for PTCA, since it has distinct advantages over balloon angioplasty alone, including the reduction of the rate of restenosis from 50% to 20% [76].

Restenosis has been fitted into the standard multifactorial model of complex disease, although classical genetic epidemiological studies (twin or extended family studies) needed to establish the heritability of the phenotype are not practical and have not been conducted. Moreover, given the known importance of anatomical factors, procedural factors and diabetes in determining the risk of restenosis, any genetic effect seems *a priori *likely to be small [77]. Despite the lack of high biological plausibility, several studies have examined the risk of restenosis in association with *ACE I/D *polymorphism.

Overall 34 studies investigating restenosis were included (Table 1 and Additional file 1). Eleven studies used PTCA with balloon angioplasty alone (PTCA-balloon) [17-26,37], 12 studies used PTCA with bare-metal stent deployment (PTCA-STENT) [29-36,43,45,46,49], nine studies used PTCA and investigated the effect of concomitant administration of ACE inhibitors (ACEi) [42-50] and two studies used directional coronary atherectomy followed by PTCA [51,52]. The data from 28 studies [17-26,29-37,42-46,49] meeting the meta-analysis eligibility criteria were synthesized (Table 1). Since the biological phenomena underlying restenosis after PTCA-balloon and PTCA-STENT are distinct (negative remodeling due to elastic recoil versus neointimal hyperplasia and inflammatory response, respectively) [78], separate meta-analyses for PTCA-balloon (11 studies included: [17-26,37]) and PTCA-STENT (12 studies included: [29-36,43,45,46,49]) were performed. Moreover, the interaction between PTCA and ACEi treatment was evaluated in the context of a third meta-analysis (five studies included: [42,44-46,49]). The findings of the studies not-included in the meta-analysis are presented in Additional file 1.

#### Main results, subgroup and sensitivity analyses

**Figure 2:**
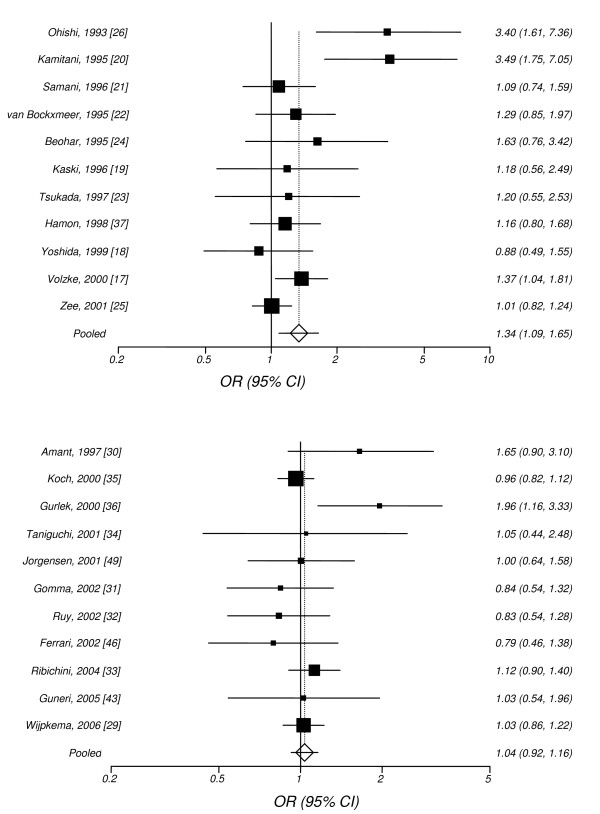
**Random effects (RE) odds ratio (OR) estimates with the corresponding 95% confidence interval (CI) of the allele contrast (*ACE D *vs. *I*) for restenosis a) after PTCA-balloon, and b) after PTCA-STENT**. The OR estimate of each study is marked with a solid black square. The size of the square represents the weight that the corresponding study exerts in the meta-analysis. The confidence intervals of pooled estimates are displayed as a horizontal line through the diamond. The horizontal axis is plotted on a log scale.

Table 2 and Figures 2a) and 2b) show the results for the association between *ACE I/D *gene polymorphism and the risk of restenosis after PTCA.

**Table 2:** Odds ratios and heterogeneity results for the genetic contrasts of *ACE I/D *gene polymorphism for restenosis a) after PTCA-balloon, b) after PTCA-STENT, and c) after PTCA and treatment with ACE inhibitors.

a)						
Genetic contrast	Population	Studies	Fixed effects OR(95%ci)	Random effects OR(95%ci)	I^2^ (%)	p-value Q-test

*D *vs.*I*	All	11	1.23(1.09–1.38)	1.34(1.09–1.65)	61	< 0.01
	Caucasians	7	1.16(1.02–1.31)	1.16(1.02–1.31)	0	0.58
	East Asians	4	1.75(1.29–2.37)	1.85(0.90–3.83)	81	< 0.01
	High quality	6	1.13(0.99–1.28)	1.13(0.99–1.28)	0	0.46
	Low quality	5	1.99(1.48–2.69)	1.95(1.21–3.14)	59	0.05
*DD *vs. (*DI+II*)	All	11	1.30(1.09–1.54)	1.42(1.07–1.91)	52	0.02
	Caucasians	7	1.20(1.00–1.45)	1.20(1.00–1.45)	0	0.57
	East Asians	4	2.29(1.38–3.80)	1.88(0.65–5.45)	70	0.02
	High quality	6	1.17(0.96–1.41)	1.19(0.96–1.47)	14	0.32
	Low quality	5	2.24(1.45–3.46)	2.15(1.12–4.10)	50	0.09
(*DD+ID*) vs. *II*	All	11	1.32(1.08–1.61)	1.36(1.05–1.76)	27	0.19
	Caucasians	7	1.23(0.98–1.54)	1.23(0.96–1.57)	10	0.35
	East Asians	4	1.78(1.12–2.82)	1.87(0.98–3.58)	44	0.15
	High quality	6	1.18(0.95–1.48)	1.18(0.92–1.51)	16	0.31
	Low quality	5	2.27(1.38–3.72)	2.21(1.33–3.66)	0	0.62
*DD *vs.*II*	All	11	1.30(1.09–1.54)	1.42(1.07–1.91)	52	0.02
	Caucasians	7	1.20(1.00–1.45)	1.20(1.00–1.45)	0	0.57
	East Asians	4	2.29(1.38–3.80)	1.88(0.65–5.45)	70	0.02
	High quality	6	1.17(0.96–1.41)	1.19(0.96–1.47)	14	0.32
	Low quality	5	2.24(1.45–3.46)	2.15(1.12–4.10)	50	0.09
*ID *vs. (*DD+II*)	All	11	0.96(0.82–1.13)	0.95(0.79–1.15)	14	0.01
	Caucasians	7	0.97(0.81–1.15)	0.95(0.77–1.18)	21	0.27
	East Asians	4	0.94(0.61–1.45)	0.93(0.55–1.55)	26	0.26
	High quality	6	0.98(0.82–1.17)	0.97(0.78–1.21)	29	0.22
	Low quality	5	0.87(0.57–1.32)	0.87(0.56–1.36)	7	0.37

b)						

Genetic contrast	Population	Studies	Fixed effects OR(95%ci)	Random effects OR(95%ci)	I^2^ (%)	p-value Q-test

*D *vs. *I*	All	11	1.03(0.94–1.12)	1.04(0.92–1.16)	25	0.21
	Caucasians	7	1.01(0.92–1.11)	1.01(0.92–1.11)	0	0.45
	East Asians	2	0.87(0.61–1.26)	0.88(0.61–1.26)	na	0.61
	High quality	6	1.02(0.93–1.12)	1.02(0.93–1.12)	1	0.41
	Low quality	5	1.05(0.84–1.31)	1.07(0.77–1.49)	52	0.08
*DD *vs. (*DI+II*)	All	11	1.05(0.92–1.21)	1.07(0.92–1.24)	7	0.38
	Caucasians	7	1.03(0.89–1.20)	1.05(0.88–1.26)	20	0.28
	East Asians	2	1.18(0.61–2.27)	1.18(0.61–2.28)	na	0.68
	High quality	6	1.04(0.89–1.21)	1.07(0.87–1.32)	32	0.20
	Low quality	5	1.16(0.82–1.65)	1.17(0.82–1.66)	0	0.56
(*DD+ID*) vs. *II*	All	12	1.01(0.87–1.16)	0.96(0.79–1.18)	30	0.16
	Caucasians	7	1.00(0.86–1.17)	1.00(0.86–1.16)	0	0.45
	East Asians	3	0.67(0.42–1.06)	0.67(0.42–1.07)	0	0.88
	High quality	6	1.02(0.88–1.19)	1.02(0.87–1.19)	0	0.43
	Low quality	6	0.93(0.67–1.30)	0.99(0.58–1.68)	51	0.07

c)						

Genetic contrast	Population	Studies	Fixed effects OR(95%ci)	Random effects OR(95%ci)	I^2^ (%)	p-value Q-test

*D *vs. *I*	Whites	3	1.10(0.81–1.48)	1.10(0.81–1.48)	0	0.42
*DD *vs. (*DI+II*)	Whites	3	1.50(0.97–2.28)	1.74(0.78–3.87)	42.8	0.17
(*DD+ID*) vs. *II*	All	5	0.85(0.52–1.38)	0.86(0.43–1.74)	31.2	0.21
	Whites	3	0.71(0.41–1.22)	0.70(0.41–1.22)	0	0.93
	East Asians	2	1.64(0.57–4.74)	1.22(0.09–16.61)	na	0.05

All studies investigating restenosis after PTCA-balloon were included in the meta-analysis. The main analysis for investigating the association between allele *D *and the risk of restenosis after PTCA-balloon relative to the allele *I*, revealed significant heterogeneity (p < 0.01) among studies and, the random effects pooled OR was significant (RE OR 1.34(1.09–1.65)). The recessive and dominant models also showed significant association (RE OR = 1.42(1.07–1.91) and RE OR = 1.36(1.05–1.76), respectively). The additive model produced significant association (RE OR = 1.42(1.07–1.91)) and the co-dominant model non-significant association (RE OR = 0.95(0.79–1.15)) as it was anticipated. Thus, *ACE I/D *polymorphism contributes to risk of restenosis under an additive model. In subgroup analysis by "race", Caucasians showed lack of significant heterogeneity (pQ = 0.58) and a marginal significance for the allele contrast (FE OR = 1.16(1.02–1.31)) whereas East Asians revealed significant heterogeneity among studies (pQ < 0.01) and non-significant association (RE OR = 1.85(0.90–3.83)).

On the contrary, in the meta-analyses investigating the risk of restenosis after PTCA-STENT or after PTCA and treatment with ACEi, there was non-significant heterogeneity among studies (pQ > 0.10) and the association was non-significant overall, for Whites and East Asians, under any genetic model.

**Figure 3:**
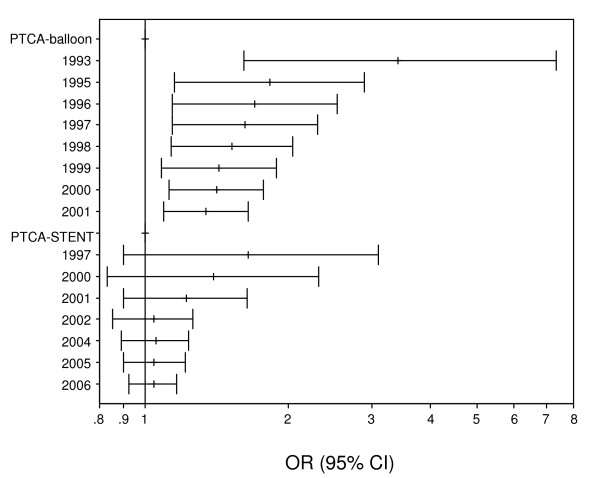
**Cumulative meta-analysis of the allele contrast (*ACE D *vs. *I*) for restenosis a) after PTCA-balloon, and b) after PTCA-STENT**. The random effects pooled odds ratio (OR) with the corresponding 95% confidence interval (CI) at the end of each year-information step is shown.

**Figure 4:**
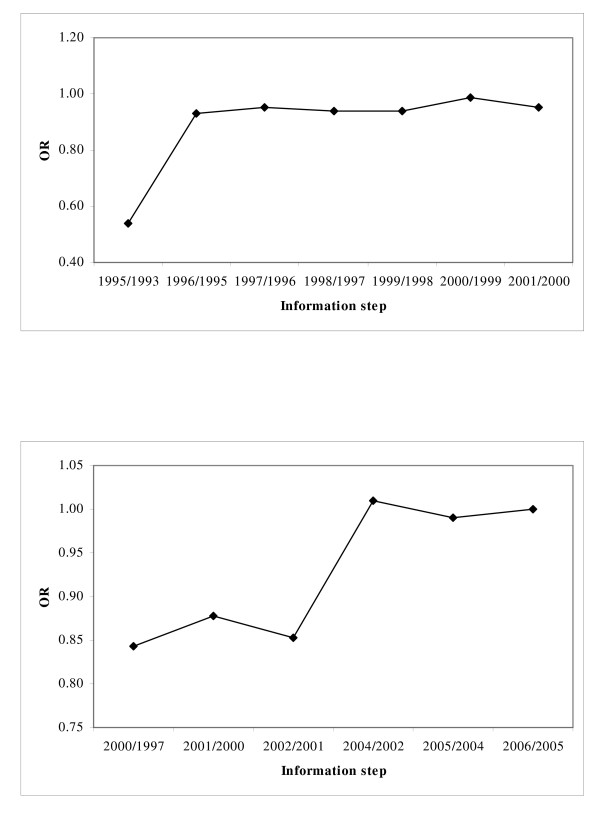
**Recursive cumulative meta-analysis of the allele contrast (*ACE D *vs. *I*) for restenosis a) after PTCA-balloon, and b) after PTCA-STENT**. The relative change in random effects pooled odds ratio (OR) in each information step (OR in next year/OR in current year) for the allele contrast is shown.

Potential Bias. The cumulative meta-analysis of the allelic contrast for restenosis after PTCA-balloon showed a trend of association as information accumulates (Figure 3). In recursive cumulative meta-analysis, the relative change in RE OR stabilized after 1996/1995 indicating that there is sufficient evidence for supporting an association (Figure 4a). In contrast, analysis for restenosis after PTCA-STENT showed that the association remained non-significant for the whole period (Figure 3). In the recursive cumulative meta-analysis, the relative change in RE OR did not stabilize in a specific OR indicating the need of more evidence for investigating the association (Figure 4b). The subgroup analysis for study quality showed lack of significant heterogeneity (pQ = 0.46) and produced non-significant association (FE OR = 1.13(0.99–1.28)) in the case of high-quality studies for PTCA-balloon. In contrast, low quality studies showed significant heterogeneity (pQ = 0.05) and significant association (RE OR = 1.99(1.48–2.69)) (Table 2). For PTCA-STENT studies, the subgroup analysis by quality did not reveal any significant associations, though significant heterogeneity across low quality studies was observed (pQ = 0.08). The Egger test for the allele contrast indicated that there is differential magnitude of effect in large versus small studies (p < 0.08).

#### Coronary artery by-pass grafting

Two small-scale studies [53,54] investigated the risk of atherosclerotic degeneration in venous grafts in association with the *ACE I/D *polymorphism, but the reported results are discrepant (Additional file 1). In a European cohort of CAD patients treated with CABG, the *ACE *genotype was an independent predictor of total and cardiac mortality in two-year follow up, explaining 17.7% of cardiac events, although a relationship of *I/D *polymorphism with graft atherosclerosis was not investigated [55].

### Conservative treatment strategies

The results are summarized in Additional file 1.

#### Anti-hypertensive therapy

Two primary prevention trials of CAD assessed the effect of *ACE I/D *on anti-hypertensive therapy and reported non-significant results overall [57,58]. The GenHAT study [57] found no differences for the primary endpoint of fatal CAD and nonfatal MI across gene-drug strata. *ACE I/D *polymorphism was found to be significant only in the subgroup of women treated with lisinopril. In the PROGRESS study that enrolled patients with history of cerebrovascular disease, there were no *ACE*-genotype specific benefits of perindopril administration on the outcome of fatal CAD and nonfatal MI [58]. A retrospective study by Marciante et al. [59] was the first study to examine the role of haplotypic variation in *ACE *gene on primary prevention of MI. None of the examined *ACE *haplotypes (capturing the *I/D *polymorphism) was associated with the risk of MI in pharmacologically treated hypertensive patients.

The effect of *ACE I/D *polymorphism and ACEi treatment on surrogate CAD outcomes was investigated by eight studies [60-67] (Additional file 1). The reported results are diverse and inconsistent, derived mainly from underpowered or non-randomised studies.

#### Lipid lowering treatment

Regarding clinical outcomes, three randomized trials failed to show any significant *ACE *genotype-statin treatment interactions in primary [68] or secondary prevention [70,71] of CAD (Additional file 1). Additionally, in the observational Rotterdam study, non-significant associations were reported overall, although a significant interaction between the *ACE *gene and the use of statins was observed in male participants [69]. Regarding the angiographic assessment of CAD progression or regression after statin treatment, the *ACE I/D *genotype was found to be a major modifier, with *DD *patients being more likely to have definite regression of coronary lesions, consistent with a greater reduction in low density lipoprotein cholesterol levels [71].

#### Cardiac rehabilitation

Two studies examined the role of *ACE I/D *polymorphism in modifying the response of physical training in aerobic and exercise capacity in CAD patients. Defoor et al. [73] found a greater beneficial effect in patients with *II *genotype than in *D *allele carriers, while Iwanaga et al. [74] reported no significant findings in their smaller study.

## Discussion

In this review, we have explored a large and varied literature and have found a wide range of quality of evidence. The studies reviewed offer inconclusive and in many cases contradictory results. The most widely investigated outcome was the restenosis post-PTCA. We conducted meta-analyses to shed some light on the contradictory results, as well as to decrease the uncertainty of the effect size of estimated risk.

The risk of restenosis following PTCA-balloon was consistent for the allele contrast, the recessive, the dominant and additive models, though the results showed significant heterogeneity. Heterogeneity may result from differences in sample selection (e.g., in age-at-onset, gender, or diagnostic criteria), in genotyping methodology (two different genotyping procedures were used), or may be due to real differences in populations (e.g., 'racial' descent) or due to interactions with other unknown risk factors [14].

The results of the meta-analysis were affected by population origin. Caucasians showed significance under the allele contrast whereas East Asians produced non-significant results. The lower frequency of the *DD *genotype in East Asian populations, coupled with the small sample size in most studies, imply that any negative conclusion could be due to low statistical power. True race-specific genetic effects could explain this pattern of results, since functional analyses of variation in *ACE *gene have indicated that different loci control ACE levels in particular 'racial' groups [79]. Nevertheless, any inconsistencies in risk effects of *ACE I/D *on restenosis between Caucasian and East Asians might be also due to race-related anatomical differences of coronary arteries, since a smaller total vessel diameter has been described for Asian populations [80].

The need for cumulative and recursive cumulative meta-analyses has already been highlighted [6]. The stability in the relative changes in ORs indicates that there is enough evidence to draw safe conclusions about the modifying effect of *ACE I/D *polymorphism in restenosis post-PTCA. However, the results of the subgroup analysis by quality [6,81] make the robustness of the main analysis questionable.

Regarding the *ACE I/D *polymorphism and the risk of restenosis after PTCA-STENT or after PTCA and treatment with ACEi, there was non-significant heterogeneity among studies and the association was non-significant overall and for all examined subgroups, under any genetic model. Despite higher biological plausibility in the context of the PTCA-STENT intervention (the renin-angiotensin-aldosterone system is considered to be more implicated in the inflammatory processes of neointimal growth underlying post-PTCA-STENT restenosis, rather than in elastic recoil remodeling following PTCA-balloon) [78], the *ACE *polymorphism was not associated with higher restenosis risk. The instability of the RE OR in the recursive cumulative meta-analysis indicated the need of more evidence to draw safer conclusions.

Our analysis showed a differential magnitude of effect in large versus small studies. Previous meta-analyses had already highlighted that the pooled estimate based on published literature, which favoured an association, was probably distorted by publication bias toward positive results [7,8]. However, our analysis was based on a substantially larger number of studies (including a total of 9945 patients vs 4631 and 3150 patients that were used in previous meta-analyses [7,8], respectively), which allowed investigation of *ACE I/D *and angioplasty interaction in the context of three clinically relevant distinct meta-analyses. The *ACE I/D *polymorphism was associated only with restenosis post-PTCA-balloon and contrary to previous findings [8], the results for this association showed significant heterogeneity. Our subgroup analysis identified ethnicity as a potential factor contributing to heterogeneity and highlighted the effect of study quality on summary estimates, which had not been previously addressed. Additionally, the studies on the *ACE I/D *and restenosis following PTCA-STENT were not significantly heterogeneous, contradicting previous findings [8], although the instability of the RE OR in the recursive cumulative meta-analysis indicated that this potential association remains an unresolved issue.

All of the analyzed studies in the meta-analysis for PTCA-STENT involved bare metal stents. Given the near-universal adoption of stenting as the default strategy for PTCA, the *ACE I/D *polymorphism can not be considered as a reliable genetic marker of restenosis after PTCA. Nevertheless, in the era of drug-eluting stents [82], late stent thrombosis emerges as a clinically important outcome that probably merits at least equivalent attention to restenosis in the design of future studies.

The treatment modalities in which *ACE *gene has been investigated as a potential modifier gene are diverse and not linked by common molecular mechanisms, thus questioning the biological rationale underlying the selection of this candidate gene. The discrepancy of the observed results regarding the clinical and surrogate outcomes of the other interventions (Additional file 1) could be due to a series of factors, including heterogeneity of enrolled cases, outcome definition variability, genotyping errors [16], limited statistical power, different study designs and variable interventions (in terms of type, dose, duration or timing). Downgrading the potential significance of *ACE I/D *polymorphism in the pharmacogenetics of CAD, none of the six studies enrolling more than 1,000 individuals [29,35,40,57,58,68] reported significant results on its respective outcomes.

Large, prospective studies with similar study designs, detailed clinical records, standardised outcome definitions, limited variability in subjects enrolled and interventions used, are needed. Moreover, if researchers can make their data on individual patients readily available, adjusted estimates for the effects of modifiers (such as age or gender) can also be analyzed.

Since the *ACE I/D *polymorphism is intronic, it is unlikely that it is functional. Despite considerable effort, the precise location of the functional polymorphism, or polymorphisms, is still unknown [83]. Future studies utilizing the HapMap tagging SNPs data, could provide useful insights, regarding the disease-associated gene haplotypes. So far, only one study [59] used the haplotype approach reporting negative results. In addition, the effect of epistatic loci interacting with *ACE I/D *remains a poorly investigated issue [7]. Elucidating the modifying effect of the renin-angiotensin-aldosterone system on response to treatment to CAD would demand a multigene haplotype approach searching for variation throughout this pathophysiological pathway [84]. With the advent of 'agnostic' genome-wide association studies, novel variants of unprecedented biological suspicion can be unravelled by properly designed and well-powered pharmacogenomic studies [85].

Cost-effectiveness analyses are crucial inputs in pharmacogenetic studies prior implementation of genetic tests in clinical practice [86]. Despite some promising initial pharmacoeconomic investigations [87], the *ACE I/D *genotype and treatment interactions in CAD are not reproducible and convincing enough to justify clinical implementation any time soon.

## Conclusion

Many studies have tried to characterize the effects of *ACE I/D *polymorphism on the response to treatment in CAD, in the context of both interventional and conservative therapeutic options for clinical and surrogate endpoints. However, the reported results so far are discrepant and inconsistent. In view of available evidence, genetic testing of *ACE I/D *polymorphism prior to clinical decision making is not currently justified. The relation between *ACE *genetic variation and response to treatment in CAD remains an unresolved issue. The results of long-term and properly designed prospective studies hold the promise for pharmacogenetically tailored therapy in CAD.

## Abbreviations

All abbreviations are defined in the text.

## Competing interests

Georgios Kitsios is Pfizer-Tufts Medical Center Research Fellow in Clinical Research.

## Authors' contributions

GK and EZ designed the study and drafted the manuscript. GK and EZ extracted the data and EZ analyzed the data. Both authors had equal contribution to the revised manuscript.

## Pre-publication history

The pre-publication history for this paper can be accessed here:



## Supplementary Material

Additional file 1 **Summary information of studies not included in the meta-analyses**. The data provided represent the extracted information from studies not considered in the meta-analyses. Click here for file
