# Supplementary material for: ACE (I/D) polymorphism and response to treatment in coronary artery disease: a comprehensive database and meta-analysis involving study quality evaluation
Source: BMC Med Genet. 2009 Jun 4;10:50. doi: 10.1186/1471-2350-10-50 (PMC2700093; doi:10.1186/1471-2350-10-50)
Supplement: Additional file 1 — Summary information of studies not included in the meta-analyses. The data provided represent the extracted information from studies not considered in the meta-analyses. [file 1471-2350-10-50-S1.doc]

| **First author, Year, Country [ref]** | **Study design, duration of FU** | **Cohort description [No of patients (M/F), Ethnicity, Mean Age (SD), inclusion criteria]** | **Outcome assessed (diagnostic criteria)***  **[Classification of outcome]** | **Intervention** | **Quality score** | **Main Findings** |
| --- | --- | --- | --- | --- | --- | --- |
|  |  |  |  | **Balloon angioplasty** |  |  |
| Hamon, 1996  France [38] | cohort  nr | 291 (nr), Caucasians, nr, CAD patients undergoing PTCA | Total occlusion  [surrogate] | PTCA-balloon | 20 | Allele contrast OR=1.71 (0.8-3.6)  Dominant model OR=0.89 (0.2-3.2)  Recessive model OR=2.98 (1.1-8.1) |
| Hertwig, 2002, Germany [27] | cohort  4-6 months | 145 (128/17), Caucasians, 59.2 (8.4), CAD patients undergoing repeat PTCA for restenotic lesions at FU angiography | Recurrent Restenosis (1)  [surrogate] | PTCA-balloon | 33 | Allele contrast OR=1.24 (0.8-2.0)  Dominant model OR=1.79 (0.8-4.2)  Recessive model OR=0.90 (0.4-2.1) |
| Mulder, 2003, Netherlands [28] | cross-sectional (PREFACE) | 52 (47/5), Caucasians, nr, CAD patients undergoing PTCA | endothelial dysfunction (2)  [surrogate] | PTCA-balloon + Pravastatin (40mg) | 23 | An ameliorating effect of pravastatin in patients with the *DD* genotype was found |
|  |  |  |  | **Angioplasty with stent deployment** |  |  |
| Gross, 2007, Germany [39] | cohort  1 year | 81 (70/11), Caucasians, 59/2 (1.4), CAD patients undergoing repeat PTCA for in-stent restenotic lesions at FU angiography | Recurrent Restenosis (1)  [surrogate] | PTCA-STENT | 31 | Allele contrast OR=1.03 (0.5-2.0)  Dominant model OR=0.51 (0.1-2.9)  Recessive model OR=1.38 (0.5-3.9) |
| Hamon, 2003, France | cohort  2 years | 1010 (813/197), Caucasians, 64 (11), symptomatic CAD patients treated with successful PTCA-STENT | Major adverse cardiac event (3)  [clinical] | PTCA-STENT | 33 | No association between *ACE*  genotype and clinical outcome was observed |
| Prisco, 2000, Italy [40] | cohort  1 year | 29 (nr), Caucasians, 60 (46-75), MI patients undergoing primary PTCA | Endothelial dysfunction (4) [surrogate] | PTCA-STENT | 34 | *ACE DD* genotype was  significantly (P=0.04) associated with an increase of PAI-1activity post PTCA [recessive model OR= 6.52 (4.8-8.2)]. No significant gene-gene interaction was observed |
|  |  |  |  | **Angioplasty with ACEi treatment** |  |  |
| Koch, 2003, Germany [47] | cohort  1 year | 612 (455/157), Caucasians, 64.3 (9.5 ), CAD patients with DD genotype undergoing PTCA | Restenosis (5)  [surrogate]  Restenosis (6)  [clinical] | PTCA-STENT + ACEi | 36 | *D* homozygotes receiving ACEi were not at a higher risk of angiographic or clinical restenosis than patients who were not treated with ACEi (p=0.55) |
| Meurice, 2001, France [48] | cohort  6 months | 79 (67/12), Caucasians, 58.9 (10.6), CAD patients with DD genotype undergoing PTCA | Restenosis (5)  [surrogate] | PTCA-STENT + Quinapril 20mg | 32 | *D* homozygotes receiving Quinapril presented a trend towards increased restenosis compared to placebo |
| Toyofyuku, 2002, Japan [50] | RCT  6 months | 204 (147/57), East Asians, 63.1 (1.0), CAD patients undergoing PTCA randomized to quinapril and placebo | Restenosis (5)  [surrogate] | PTCA-STENT + Quinapril 20mg | 30 | No significant interaction between *ACE* polymorphism and quinapril treatment was observed on the risk of restenosis. No significant gene-gene interaction was observed |
|  |  |  |  | **Coronary Atherectomy** |  |  |
| Haberbosch, 1997, Germany [51] | cohort  12-18 months | 104 (91/13), Eur Whites, 60.2 (nr), CAD patients undergoing DCA and PTCA | Restenosis (5)  [surrogate] | DCA-PTCA-STENT | 26 | Allele contrast OR=2.19 (0.9-4.8)  Dominant model OR=4.78 (0.6-38.3)  Recessive model OR=2.33 (0.8-6.7) |
| Canosi, 2004, Italy [52] | Cohort  6.3 (1.5) months | 113 (103/10), Eur Whites, nr, CAD patients undergoing DCA and PTCA | Restenosis (5)  [surrogate] | DCA-PTCA-STENT | 29 | Allele contrast OR=0.90 (0.4-1.9)  Dominant model OR=1.17 (0.2-5.7)  Recessive model OR=0.71 (0.2-2.4) |
|  |  |  |  | **CABG** |  |  |
| Dayi, 2005, Turkey [53] | cross-sectional | 87 (71/16), Turks, 64.3 (7.3), CAD patients treated with CABG before >5 years undergoing angiography for symptoms | Venous graft atherosclerosis (7)  [surrogate] | CABG | 28 | Allele contrast OR=2.41 (1.3-4.5)  Dominant model OR=3.76 (1.2-12.2)  Recessive model OR=3.75 (1.3-11.3) |
| Ortlepp, 2001, Germany [54] | cohort  88 (52) months | 101 (86/15), Caucasians, 64.0 (8.1), CAD patients after CABG presenting for angiography due to angina pectoris | Venous graft atherosclerosis (8)  [surrogate] | CABG | 33 | The *ACE I/D* polymorphism was not associated with by-pass degeneration |
| Volzke, 2002, Germany [55] | cohort  2 years | 247 (202/45), Caucasiana, 64.9 (nr), CAD patients undergoing CABG | - total mortality  - cardiac mortality or need for recurrent revascularization  [clinical] | CABG | 35 | *ACE I/D* genotype is an independent predictor of midterm total mortality after CABG [Mortality rate: *II*=0%, *ID*=11.2%, *DD*=14.1%, p<0.05]. The *ACE I/D* genotype was also an independent predictor of the secondary end-point [cumulative cardiac event incidence: *II*=5.8%, *ID*=9.4%, *DD*=30.3%, p<0.005] |
| Voors, 2004, Netherlands [56] | cohort  12 months | 82 (71/11), Caucasians, 61.5 (1.3), CAD undergoing CABG | endothelial dysfunction (9)  [surrogate] | CABG +quinapril (40mg) | 27 | quinapril completely restored the decreased vascular response in *DD*-genotype patients to the same level as *II/ID* genotype patients, while no effect of quinapril was demonstrated in the *II/ID*-genotype patients. Quinapril also prevented the increase in plasma ACE activity after CABG, especially in *DD* homozygotes |
|  |  |  |  | **Anti-hypertensive therapy** |  |  |
| Arnett, 2005, USA [57] | RCT (GenHAT)  6 years | 37939 (20154/ 17785), mixed, 66.8 (7.7), high-risk hypertensive patients | fatal CAD and nonfatal MI  [clinical] | chlorthalidone, lisinopril, amlodipine, and doxazosin | 43 | No evidence that the risk of CAD differed across gene-drug strata. The absence of gene-treatment interactions persisted for all ethnic and age subgroups. Only sex-gene-treatment interaction was obvious (Women with the *DD* genotype treated with lisinopril versus chlorthalidone or amlodipine had an increased risk of CAD [OR= 1.32, 95%CI(1.02 to 1.69)], whereas men had a lower risk). |
| Harrap, 2003,USA [58] | RCT (PROGRESS)  4 years | 5688 (3961/1727), mixed, 63.3 (9.1), history of Cerebrovascular disease (stroke or TIA) within the previous 5 years and no clear indication for or contraindication to treatment with an ACEi. | fatal CAD and nonfatal MI  [clinical] | perindopril (4 mg) | 34 | no evidence that the beneficial effects of perindopril for prevention of CAD differed among the genotypes |
| Marciante 2007, USA [59] | case-control,  retrospective | 349 (210/139), mixed, 64.5 (10.5), MI survivors retrospectively collected, being on anti-hypertensive therapy on the index day (day of admission for MI) | Occurrence of MI  [clinical] | Anti-hypertensive therapy | 38 | None of the examined common *ACE* haplotypes (including the *I/D* polymorphism) was associated with the risk of MI in pharmacologically treated hypertensive patients. |
| Pinto, 1995, Germany [60] | RCT (CATS)  1 year | 96 (70/26), Caucasians, 58.7 (9), first anterior MI treated with streptokinase infusion | post-MI left ventricular remodelling  [surrogate] | captopril (75mg) | 27 | Captopril treatment in the *DD*-genotype group blunted the significant increase in left ventricular end-systolic volume index observed in the placebo-treated *DD*-genotype group |
| Zee, 2002, USA [61] | RCT (HEART)  90 days | 265 (207/58), Caucasians, 60.1 (12.5), first anterior MI | post-MI left ventricular remodelling  [surrogate] | ramipril (1.25-10mg) | 36 | no evidence for an association of the *ACE I/D* polymorphism with the risk of left ventricular remodelling post-MI in the presence of ramipril |
| Kennon, 2001, UK [62] | cross-sectional | 301 (225/76), mixed, 61.3 (10.8), Braunwald class 3B UA criteria | troponin release in non-ST elevation acute coronary syndromes [surrogate] | ACEi | 26 | No association between troponin release, *ACE* genotype and pre-treatment with ACEi |
| Prasad, 2000, USA [63] | cross-sectional | 56 (nr), mixed, 52 (1), patients undergoing coronary angiography | endothelial dysfunction (10)  [surrogate] | enalaprilat (0.2 mg iv) | 32 | Significant Acetylcholine-mediated microvascular dilation improvement was only observed in patients with the *DD* and *ID* genotypes, who had a 20.10% (P=0.01) and 18.8% (P<0.01) further increase in coronary blood flow with enalaprilat, respectively, compared with a nonsignificant 2.8% (P=0.8) decrease in flow in those homozygous for the I allele |
| Trevelyan, 2004, UK [64] | cohort  5 months | 49 (49/00), Caucasians, 63.5 (1.8), men with normal left ventricular function and stable angina awaiting CABG free of ACEi, AT1 receptor antagonist or b-blocker treatment | endothelial dysfunction (11)  [surrogate] | enalapril (10mg) or losartan (50mg) | 34 | Enalapril and losartan significantly improved brachial artery flow mediated dilatation in all groups, with a graded response noted among the genotypes: DD (3.1 (1.1)%, p = 0.02 vs baseline), DI 5.5 (3.1 (1.3)%, p = 0.024 vs baseline), II (7.1 (1.1)%) (p = 0.005 vs baseline). |
| Pedersen, 1997, Denmark [65] | cohort  12 months | 56 (47/9), Caucasians, 60.5 (7.5), MI survivors randomized with moderate left ventricular dysfunction | Endothelial dysfunction (12) [surrogate] | Trandolapril (4 mg) | 26 | No significant differences in the effects of trandolapril treatment among the genotypic groups were observed, regarding the fibrinolytic variables and ACE activity. |
| Jeron, 2001, Germany [66] | cross-sectional | 609 (532/77), Caucasians, 56.2 (0.7), MI survivors | QT segment dispersion  [surrogate] | ACEi | 31 | The *ACE DD*-genotype was associated with longer QT dispersion in myocardial infarction patients (103.0 (4.6) ms vs 81.9 (4.5) ms in the *II* group, P<0·001), and this association was unaffected by treatment with ACEi |
| Steeds, 2002, UK [67] | cohort  10 days | 149 (107/42), Caucasians, 63.5 (11.5), MI survivors | Heart Rate Variability  [surrogate] | ramipril (2.5mg) | 31 | No differences were observed in Heart Rate Variability response to ACE inhibition following MI between the *ACE* genotypes |
|  |  |  |  | **Lipid lowering therapy** |  |  |
| Maitland van der Zee, 2007, Netherlands [68] | RCT (GenHAT)  6 years | 4741 (2434/2307), mixed, 66.6 (7.7), high-risk hypertensive patients | fatal CAD and nonfatal MI  [clinical] | pravastatin (40mg) | 41 | no evidence that the *ACE ID* genotype was a major modifier of the efficacy of pravastatin in reducing the risk of CAD. No sex-gene-drug or race-gene-drug interactions were observed. |
| Maitland van der Zee, 2004, Netherlands [69] | cohort  nr | Nr (nr), Caucasians, nr, subjects with total cholesterol ≥6.5 mmol/l or using statins in the population-based Rotterdam study | CAD event (13)  [clinical] | statins (nr) | 23 | No significant interaction between the use of statins and *ACE* genotype on the occurrence of CAD events. A significant sex-gene-drug interaction was observed [Synergy Index in men was 7.41 (95% CI 1.17–46.8)]. |
| Bray, 2001, USA [70] | RCT (CARE)  5 years | 385 (331/54), Caucasians, 59.2 (9.7), MI survivors with total cholesterol <6.2 mmol/L and LDL-C of 3.0-4.5 mmol/l | fatal CAD and nonfatal MI  [clinical] | pravastatin (40mg) | 30 | Pravastatin’s effects on reducing the primary end point were unrelated to *ACE* genotype.However, the greatest effect of pravastatin on reducing the primary end point was in patients with *PlA1,A2* who were *ACE D* “positive” (either *ACE ID* or *DD*). The relative benefit of pravastatin therapy was a 45% risk reduction in patients who had the *PlA1,A2 1 ACE D* positive genotype (p=0.09). |
| Marian, 2000, USA [71] | RCT (LCAS)  2.5 years | 429 (349/80), Caucasians, 58.8 (7.8), one coronary lesion causing 30% to 75% diameter stenosis and LDL-C of 115–190 mg/dl | - Progression or regression of coronary atherosclerosis (14)  [surrogate]  - Clinical events (15) [clinical] | fluvastatin (40mg) | 31 | *DD* homozygotes were less likely to have definite progression (14%) and more likely to have definite regression (24%) compared with those with *ID* (32% progression and 17% regression) or *II* (33% progression and 3% regression) genotypes (p=0.023).The distribution of the clinical events among the genotypes was not significantly different |
| Talmud, 1995, UK [72] | cohort (STARS)  3 years | 73 (nr), Caucasians, nr, nr | Progression or regression of coronary atherosclerosis (16) [surrogate] | diet and cholestyramine | 20 | There was overall regression in the group with the genotype *II* or *ID*, while in the group with the genotype *DD*, there was slight progression of coronary atherosclerosis (p=0.03) |
|  |  |  |  | **Cardiac rehabilitation** |  |  |
| Defoor, 2006, Belgium [73] | cohort  3 months | 933 (857/76), Caucasians, 56 (0.3), patients with CAD who had achieved evident exhaustion during graded cycle ergometer testing before and after three months of physical training | aerobic power response to physical training (17)  [surrogate] | physical training (3 sessions weekly) | 24 | The covariate adjusted aerobic power response to training was greater in patients with *ACE II* than in *D* allele carriers (p=0.047) |
| Iwanaga, 2005, Japan [74] | cohort  3 months | 168 (143/25), East Asians, 59 (1.0), MI survivors | exercise capacity response to physical training post-MI (18)  [surrogate] | physical training (4-5 sessions weekly) | 28 | No significant differences in the increases in exercise capacity parameters were observed among the 3 genotype groups after 3-month physical training program |

Outcome definition criteria: 1. Restenosis defined as >50% progression of the residual stenosis at FU angiography compared with the findings immediately after PTCA, 2. Endothelial dysfunction defined as coronary endothelium dependent vasomotion after intracoronary infusions of acetylcholine, 3. Major adverse coronary event defined as death, MI, UA and coronary revascularisation, 4. Endothelial dysfunction defined as plasminogen activator inhibitor activity post-PTCA, 5. Restenosis defined as diameter stenosis >50% at FU angiography, 6. Clinical restenosis defined as need for target vessel revascularization due to symptoms or signs of ischemia in the presence of angiographic restenosis over one year after the intervention, 7. Venous graft atherosclerosis defined as total occlusion of venous graft, 8. Venous graft atherosclerosis defined by Gensini by-pass degeneration score, 9. Endothelial dysfunction defined as maximal vasoconstriction to angiotensin II, 10. Endothelial dysfunction was defined as coronary vasomotor responses to acetylcholine and sodium nitroprusside, 11. Endothelial dysfunction was defined as brachial artery flow mediated dilatation, 12. Endothelial dysfunction was defined as tissue plasminogen activator, tissue plasminogen activator inhibitor-1 and ACE activity, 13. CAD event defined as MI (fatal and non-fatal), CABG, PTCA and all coronary mortality (including mortality caused by ischemic heart disease, sudden (cardiac) death, ventricular fibrillation and congestive heart failure), 14. Definite progression was defined as ≥1 qualifying lesion with minimum lumen diameter decrease ≥0.4-mm, including new total occlusions and no qualifying lesion with minimum lumen diameter increase ≥4 mm. Definite regression was defined as ≥1 qualifying lesion with minimum lumen diameter increase ≥0.4 mm, no qualifying lesion with minimum lumen diameter decrease ≥0.4 mm and no new total occlusion, 15. Clinical events defined as MI, hospitalized UA, PTCA, CABG, all-cause mortality and cardiovascular mortality, 16. Coronary atherosclerosis progression or regression defined as change in the mean absolute width along each segment and the change in the minimal absolute width for each segment, 17. Aerobic power response was defined as peak oxygen uptake (ml/min), 18. Exercise capacity response was defined as peak work rate, peak oxygen uptake (ml/min) and the slope of the minute ventilation – carbon dioxide production relationship

Abbreviations: CAD: coronary artery disease, MI: Myocardial infarction, PTCA: percutaneous transluminal coronary angioplasty, CABG: coronary artery by-pass grafting, UA: unstable angina, LDL-C: low density lipoprotein cholesterol, RCT: randomized controlled trial, TIA: transient ischemic attack, FU: follow-up, DCA: directional coronary atherectomy, nr: non-reported
